# Development and interval testing of a naturalistic driving methodology to evaluate driving behavior in clinical research

**DOI:** 10.12688/f1000research.9150.2

**Published:** 2016-09-15

**Authors:** Ganesh M. Babulal, Aaron Addison, Nupur Ghoshal, Sarah H. Stout, Elizabeth K. Vernon, Mark Sellan, Catherine M. Roe

**Affiliations:** 1 Charles F. and Joanne Knight Alzheimer’s Disease Research Center, Department of Neurology, Washington University School of Medicine, St. Louis, USA; 2 University Geographic Information System, Washington University, St. Louis, USA

**Keywords:** naturalistic driving, interval testing, geographic information system, global positioning systems, in-vehicle technology

## Abstract

**Background**: The number of older adults in the United States will double by 2056. Additionally, the number of licensed drivers will increase along with extended driving-life expectancy. Motor vehicle crashes are a leading cause of injury and death in older adults. Alzheimer’s disease (AD) also negatively impacts driving ability and increases crash risk. Conventional methods to evaluate driving ability are limited in predicting decline among older adults. Innovations in GPS hardware and software can monitor driving behavior in the actual environments people drive in. Commercial off-the-shelf (COTS) devices are affordable, easy to install and capture large volumes of data in real-time. However, adapting these methodologies for research can be challenging. This study sought to adapt a COTS device and determine an interval that produced accurate data on the actual route driven for use in future studies involving older adults with and without AD.

**Methods**: Three subjects drove a single course in different vehicles at different intervals (30, 60 and 120 seconds), at different times of day, morning (9:00-11:59AM), afternoon (2:00-5:00PM) and night (7:00-10pm). The nine datasets were examined to determine the optimal collection interval.

**Results**: Compared to the 120-second and 60-second intervals, the 30-second interval was optimal in capturing the actual route driven along with the lowest number of incorrect paths and affordability weighing considerations for data storage and curation.

**Discussion**: Use of COTS devices offers minimal installation efforts, unobtrusive monitoring and discreet data extraction.  However, these devices require strict protocols and controlled testing for adoption into research paradigms.  After reliability and validity testing, these devices may provide valuable insight into daily driving behaviors and intraindividual change over time for populations of older adults with and without AD.  Data can be aggregated over time to look at changes or adverse events and ascertain if decline in performance is occurring.

## Background

Motor vehicle crashes (MVC) are a leading cause of injury among older adults (586 daily) in the United States ^1^ and MVC deaths have steadily climbed over the past decade, along with an increase in crash risk with each year ^2^. Coupled with the growth of the aging population and the increasing prevalence of dementias like Alzheimer disease (AD), being able to predict when driving performance will decline may prevent crashes and deaths among older adult drivers and others who share the roadway ^3– 5^.

To this end, our research program seeks to better characterize the driving behaviors of older adults and predict the onset of driving difficulties so that we can implement appropriate interventions to maintain safety and prolong driving life ^6^. We are particularly interested in the association between preclinical AD and driving.

Road tests and driving simulators are the most common and dominant measures used to assess driving performance and determine road safety ^7^. Both methods have proven reliable and valid in evaluating poor driving performance and estimating crash risks for older drivers ^8, 9^. However, driving is an overlearned task and controlled conditions like the road test and simulator may not reflect driving as it occurs on a daily basis or expose errors made by experienced or cognitively-normal drivers outside of these controlled conditions ^10^. Other limitations of both methods include rater subjectivity, anxiety (poorer performance), Hawthorne effect, dedicated single site measures, simulator sickness and high equipment cost, maintenance, and programming ^10– 13^.

Due to these limitations, we sought to find an objective, cost-effective method that would allow us to assess future research of driving performance longitudinally on a daily basis among hundreds of older adults in the actual environments that they drive, something that has been unavailable until now. This manuscript describes the first step in our work to adapt a commercial global positioning data acquisition system (GPDAS) and develop a methodology to evaluate driving performance. This technology is capable of collecting data at a constant rate over any determined time. However, due to the cost of data storage and greater programming time with larger volumes of data, we sought to determine the “optimal” time interval for accurate data collection using GPDAS devices.

## Methods

### GPDAS device

The GPDAS device (G2 Tracking Device ^TM^ Azuga, Inc) is compact (length = 1.7”, width = 1.8”, height = 1”, weight = 1.1 ounce), plugs into the on-board diagnostic systems port (OBDII) and uses the vehicle’s own battery to supply the 12 volts required to function. Only vehicles manufactured in 1996 or later are compatible with the device. The device’s wireless capabilities include use of third generation mobile phone network (3G), jamming detection, Bluetooth, internal antenna and Firmware-Over-The-Air update for configuration of device firmware. Its global positioning system (GPS) capability includes a 56-channel receiver with a 4-Hertz acquisition rate, accuracy of 2.5 meters circular error probable (CEP) and integrated anti-jamming capability. Finally, it has a tri-axial (X, Y, Z) accelerometer with 8–13 bits of resolution on each axis. The accelerometer can detect and report changes in acceleration over +/- 16 g-force and the data can be reported at a rate ranging from 1 to 24 Hertz.

### Standard protocol approvals, registrations, ethics and consents

The GPDAS device sends data at intervals of 30, 60, or 120 seconds, which shows the exact location, speed, and date/time at each interval. The optimal time interval would accurately represent the route traveled using the minimum number of data points possible in order to minimize cost and extraneous data collection. The data collected did not contain any personal or identifying information about the drivers. Ethical permission to conduct this study was sought and received via expedited review from the Washington University Human Research Protection Office who determined that this is a non-human subjects study (201412024). Informed consent was obtained from all drivers who participated in this study.

### Data acquisition

After being plugged into the OBDII port, the GPDAS device extracts the signal from the vehicle speed sensor (VSS), which measures the transaxle speed, also known as the wheel speed. The VSS is the reference speed that the majority of a vehicle’s systems rely upon to achieve their specific functionality. For example, the Engine Control Module uses the VSS signal to modify engine functions and initiate specific diagnostic routines, while the variable assist power steering system uses it to regulate power steering pressure for assistance at slow speeds. The speed displayed on the speedometer is generally greater than the actual VSS signal, ranging anywhere between 1–3 mph more. The VSS signal, which the GPDAS device uses, is the most accurate reflection of the vehicle speed. Installation takes less than one minute, and once plugged in, the device accesses available satellites for orientation and then begins simultaneously transmitting data to secured servers using available cell phone towers. These data can be then accessed online in real-time or stored in a database for retrieval at a later date. If a vehicle is driven in areas where cellular signal is lost, data continue to be collected and is then re-transmitted when a stronger signal is established. When a vehicle is turned off, the device enters sleep mode but sends a signal every four hours to indicate the ignition is off but the device is still functioning. When a vehicle is turned on, the device immediately begins sending data at a specified time interval. A standard set of data is obtained during a trip, which is specified as the time between when the ignition is turned on and off. These variables include time and date stamp, drive time, idling time, miles, latitude and longitude, speeding over posted speed limit, hard braking, sudden acceleration and an alert if the device was unplugged and plugged back in. Since the device is powered by the vehicle’s battery, if the battery starts to drop below the required 12V, the device sends out a series of alerts indicating insufficient power and will stop transmitting if the power drops below 10V. Additionally, the device will detect problem codes that the vehicle’s computer may send out (e.g. check engine light indicating oxygen sensor requires replacement).

### Outcome

**Figure 1.**
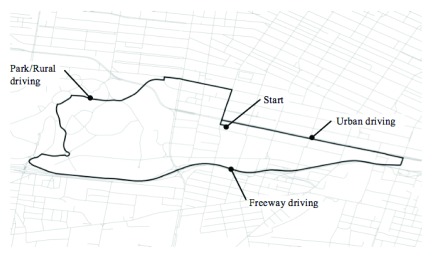
Structured driving route.

A structured driving course of approximately seven miles ( Figure 1) was designed to represent various real-world driving conditions with a comprehensive mix of stoplights, stop signs, right and left hand turns and merging into traffic. The route began at an office complex in an urban setting and continued several blocks east following a divided boulevard. Drivers then turned south to merge onto a freeway. The freeway section of the route provided driving conditions and associated data logging for highway speeds. Drivers then exited into a large park where the designated route was designed to simulate more rural driving conditions. The park section also allowed for more nuanced driving such as roundabouts where data interval logging could be analyzed for correctness to the real-world, and to simulate driving events such as a U-turn or missed turn. Finally, the drivers exited the park and returned to the office complex starting point driving on surface streets with traffic to simulate additional urban conditions.

A map of the route was provided to each member of the driving research team, as well as the turn-by-turn driving directions. Drivers did not navigate the route prior to data collection. All GPDAS data were logged into daily csv files with results uploaded to a secured server by Azuga. Automation scripts were used to validate files and copies stored on a secured server. A secured file transfer protocol was designed and automated to transfer the log files from Azuga’s server to our servers on a daily basis.

### Protocol

Three healthy subjects drove a single course in three different vehicles. The drivers negotiated the course at three different time intervals (30 seconds, 60 seconds, 120 seconds), and at three different times of day, morning (9:00-11:59AM), afternoon (2:00-5:00PM) and night (7:00-10pm). In order to minimize bias associated with the order of driving combinations and day, the time intervals and time of day were randomized for all drivers. Depending on the time of day, data were collected over several days, including weekdays and weekends. Each driver yielded nine sets of data (i.e., all possible combinations of time interval and time of day). The device remained installed in the vehicle without removal until each driver completed the set of routes.

### Statistical analyses

**Figure 2.**
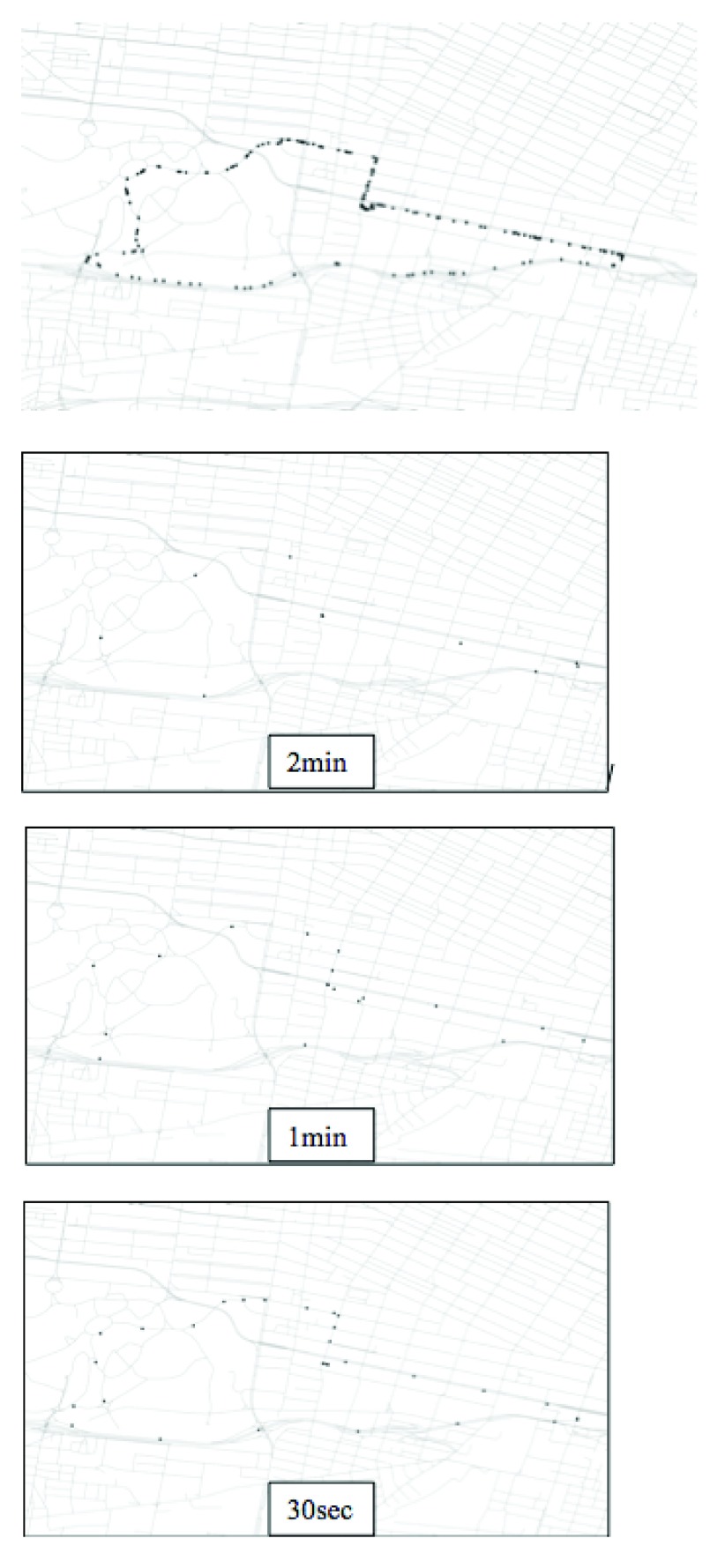
GPS points collected.

Data were logged into files (csv) stored in a secure Amazon S3 folder. Data were downloaded in bulk and sorted into folders based on the respective driver IDs. Secondary sorting was done for time interval and filtered for spurious data points. All data were imported to ArcGIS Desktop 10.2 software (Environmental Systems Research Institute, Redlands, CA, USA) and plotted on a map using the latitude and longitude coordinates logged by the GPDAS device ( Figure 2).

**Figure 3.**
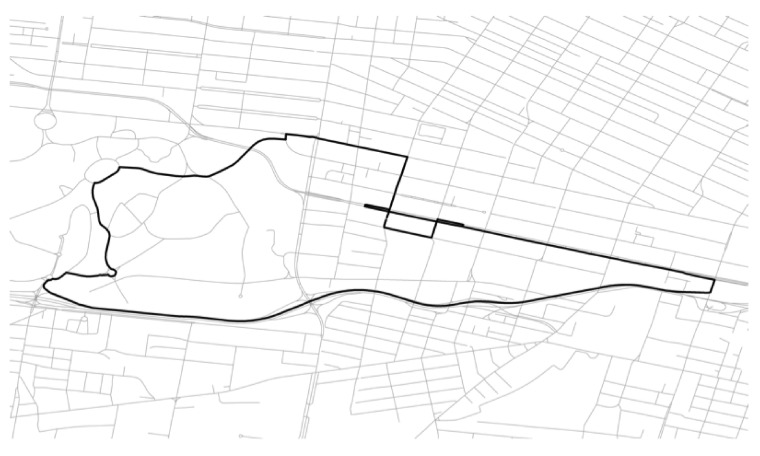
1 minute AM routes.

Each dataset was queried for a specific time interval, such as 30 seconds. The resulting dataset was used as an input for the Network Analyst extension of ArcGIS. A base road network (edge network) was also loaded into the tool for the routing algorithm. Routing algorithms use an impedance to determine “cost” of travel on the network, but are often defined in terms of time needed to traverse a given section of the network or distance needed to travel the network segment. The network impedance was defined by time of travel on the base network. The routing algorithm processed all coordinate data from each driving circuit, creating a line representing the path traveled during data collection. These data were then visualized in ArcGIS as lines ( Figure 3).

Each of the time intervals was evaluated for best fit to the base road network. Best fit was determined by comparing the route generated by the routing algorithms to the actual true route of the course. ArcGIS was used to conduct spatial comparisons between the routes driven and the real-world road course or “correct” route to determine “best” fit. The results of this analysis were used to determine the preferred data collection interval for the device.

## Results

Determining the optimal interval collection for a Global Positioning Data Acquisition System Vehicle Name: Participant ID via Vehicle Make and Model; Vehicle Speed: Vehicle speed (miles per hour); Latitude: The angular distance of a place north or south of the earth's equator (degrees); Longitude: The angular distance of a place east or west of the earth's equator (degrees); Event Type: Type of event coded by the GPDAS device; Timestamp: Date (mm/dd/yyy) and Time (hh:mm:am/pm); Odometer Reading: Vehicle odometer reporting the number of miles. Click here for additional data file. Copyright: © 2016 Babulal GM et al. 2016 Data associated with the article are available under the terms of the Creative Commons Zero "No rights reserved" data waiver (CC0 1.0 Public domain dedication).

The 30-second collection interval was determined by ArcGIS Desktop 10.2 software to have the strongest “goodness of fit” and lowest number of incorrect paths traveled compared to the one and two minute data collection intervals (See Figure 2). The mapped routes were displayed and symbolized by time interval for the main visualization product. In addition to identification of the preferred time interval for data collection, the visualization process also revealed data artifacts and incorrect routing of the base network. The source of these errors was explained primarily by the interval of data collection. For instance, if the data collection interval is too large (e.g. 120 seconds), the driver may travel through several turns before the next valid data point is logged. This absence of specific data points (e.g. found in 120 seconds interval) to guide the routing algorithm may lead to incorrect assumptions, resulting in incorrect path of travel produced.

**Figure 4.**
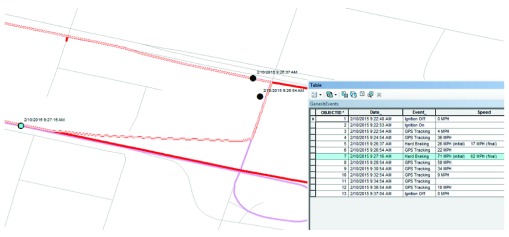
Incorrect routes.

As Figure 4 illustrates on the two-minute interval, an incorrect line was generated (zigzag line) due to lack of data about the actual route (red line). Other events, such as hard braking, can also add intermediate data points to aid in the process, but these points are unpredictable and cannot be relied upon for routing protocols since any stimulus from the external environment can impact driving behavior and trigger an event.

## Discussion

This study investigated the optimal time interval for data collection using a GPDAS device to accurately capture a driven route while weighing the considerations of cost associated with data storage and post-processing efforts. The 30-second interval was determined to be the most accurate based on goodness of fit and was affordable for our research program. Technological innovations have led to faster processors and ability to gather greater volumes of data. Yet, the challenges required to analyze big data include large statistical and computational costs, incidental homogeneity, noise, and an inherent requirement to develop newer, robust statistical models to deal with larger sample sizes ^14, 15^. Further, given a stricter funding climate, researchers working with clinical populations cannot afford the time and cost to collect, process and analyze continuous data using existing naturalistic research paradigms. Some studies use in-vehicle data recorders that require hours of installation and extensive modification of participants’ vehicle ^8^. Others use in-vehicle cameras which may modify driving behavior and also require hours of post-processing and extensive rater training ^13, 16^. Larger studies that collect hundreds of hours of data may require participants to regularly return to the study site and have the data from their vehicle downloaded ^17^. Studies using smart phone applications require participants to charge phones, turn the phone on and off and to remember to bring it in the vehicle when driving, thereby elevating participant burden ^18^.

As a whole, driving research and crash prevention research is shifting toward the use of naturalistic methodologies for evaluating driving performance ^19– 22^. Development and interval testing of a naturalistic driving methodology to evaluate driving behavior is required to measure real world driving conditions and responses ^23^ in a variety of clinical populations. The methodology presented here implements a non-obtrusive device installed in the OBDII port of a vehicle. The device stores locational data in the form of latitude and longitude at each time sampled, as well as driving behaviors that may occur at any time, such as hard braking, speeding, and vehicle on/off events. Since all data are tied to a spatial location, it is possible to understand the “place” of where data have been collected.

It is important to note that driving events such as “what happened at this exact moment or day?” should not be singled out. The inherent value of longitudinal data collection is to collect data to better understand changes over time for an older adult driver that may be otherwise hidden from observation. The true potential of this methodology is that data gathered could be linked to other databases to answer a number of questions. One can link weather and meteorological databases to understand the impact of the weather on driving patterns and Department of Transportation databases on road construction to explore how roadwork influences driving navigation. Driving behavior for clinical populations could also be evaluated in a pre-post design for patients who have had medication changes, surgeries, stroke, undergoing chemotherapy or radiation, a diagnosis of seizures or a range of neurological conditions that ultimately impact driving for a brief or longer period of time. Naturalistic driving research has the potential to study and aid the management of driving behavior of older adults with chronic neurological disease like dementia. The long-term goal of our program is to model driving behavior and driving risk of older adults using this naturalistic driving data to identify driving decline over time and develop educational interventions to improve driving performance, decrease vehicle crash risk while driving and structure driving retirement for older adults with a higher risk for MVCs.

## Data availability

The data referenced by this article are under copyright with the following copyright statement: Copyright: © 2016 Babulal GM et al.

Data associated with the article are available under the terms of the Creative Commons Zero "No rights reserved" data waiver (CC0 1.0 Public domain dedication). 

*F1000Research*: Dataset 1. Determining the optimal interval collection for a Global Positioning Data Acquisition System, 10.5256/f1000research.9150.d128877 ^24^

## Consent

Informed consent was obtained from all drivers who participated in this study from Washington University Human Research Protection Office.
