# Supplementary material for: Development and interval testing of a naturalistic driving methodology to evaluate driving behavior in clinical research
Source: F1000Res. 2016 Sep 15;5:1716. Originally published 2016 Jul 15. [Version 2] doi: 10.12688/f1000research.9150.2 (PMC5063038; doi:10.12688/f1000research.9150.2)
Supplement: Peer review history

# Peer review: Referee response for version 2

**DOI:** 10.5256/f1000research.10338.r16901

**Published:** 2016-10-11

**Authors:** David K. Johnson

**Affiliations:** 1 Department of Psychology, Center for Computational Biology, University of Kansas, Lawrence, KS, USA

The paper looks much better. My concerns have been satisfied.

I have read this submission. I believe that I have an appropriate level of expertise to confirm that it is of an acceptable scientific standard.

# Peer review: Referee response for version 1

**DOI:** 10.5256/f1000research.9846.r15049

**Published:** 2016-08-30

**Authors:** Monique M. Williams

**Affiliations:** 1 VITAS Healthcare, St. Louis, MO, USA

The authors describe the use of a commercial-off-the-shelf (COTS) device to ascertain which interval provided the most accurate data regarding the route driven. The objective is to determine the optimal interval that would be applied to future research studies involving individuals with and without Alzheimer’s disease.

 A total of three participants drove a single 7-mile driving course. The course was designed to reflect real world driving conditions. Participants drove in different cars, at different times of day (morning, afternoon, and night), and different intervals (120-second, 60-second, and 30-second). The three participants were healthy and cognitively normal. 

 The datasets were analyzed. The preferred data collection interval was determined based on the results of best fit. Best fit was operationalized as the route generated by the routing algorithm as compared with the actual route driven. The analyses revealed that the 30-second interval provided the optimal (best fit) characterization of the route. 

 The ability to use a COTS device to assess driving skills has considerable public health implications in the context of an aging nation. The study is a critical initial step in determining the utility of the COTS device for prospective research studies. With the inherent limitations of driving simulators and road testing, the use of the device as described provides a cost-effective option that more closely emulates real world driving experiences.

I have read this submission. I believe that I have an appropriate level of expertise to confirm that it is of an acceptable scientific standard.

# Peer review: Referee response for version 1

**DOI:** 10.5256/f1000research.9846.r15332

**Published:** 2016-08-01

**Authors:** David K. Johnson

**Affiliations:** 1 Department of Psychology, Center for Computational Biology, University of Kansas, Lawrence, KS, USA

Three subjects drove a single course in different vehicles at different intervals (30, 60 and 120 seconds), at different times of day, morning (9:00-11:59AM), afternoon (2:00-5:00PM) and night (7:00-10pm). The nine datasets were examined to determine the optimal collection interval. The 30-second interval was reported as optimal. Time of day was apparently uninformative.

 I think this paper could make a significant contribution to a burgeoning and important literature. How to measure driving in older adults is a complex issue. If there were valid processes and instrumentation for the meaningful and accurate measurement of safety-relevant driver outcomes in older adults, this contribution would be huge. However, I had a hard time following the results and conclusions drawn by the investigators.

 I found that most of this manuscript read like a proposal and it editing was seemingly incomplete. As it currently reads, there are significant revisions that need to be made to improve its intelligibility. For instance I had a very hard time finding any real or intelligible outcomes in the results section. The only results I could find were in the abstract (What does it mean to "capture the actual route driven along with the lowest number of incorrect paths") and then again buried in the discussion section properly. Is there really no intelligible data in the results section? Further, it is opaque to me why 30 seconds is preferred - what data supports the 30-second interval and the investigators’ reasoning behind this the acceptance of this interva? If it's GIS type data, why isn't reported and interpreted? There is an oblique reference to a GOF but where is the table and logic behind the decision to adopt one model over another? More detail and logic is needed to make the authors' writing, logic and conclusions more clear. At the end of the manuscript I felt that the abstract's conclusion that 30 seconds was an optimal time for data collection is unsupported substantively.

I have read this submission. I believe that I have an appropriate level of expertise to confirm that it is of an acceptable scientific standard, however I have significant reservations, as outlined above.

# Response

**Published:** 2016-09-07

**Authors:** Ganesh Babulal

**Affiliations:** Washington University School of Medicine, USA

Dr. Johnson,

 Thank you for your review and comments. They were helpful in our revision. This brief method article sought to determine which interval was more accurate/optimal given the available choices using GIS data by adopting a commercially off the shelf GPS device. We designed a study to examine if the 30 seconds collection interval was more accurate than the 60 and 120 seconds. We found that 30 seconds was indeed more accurate across the three drivers, days and times. Based on your feedback, we relocated the main finding at the beginning of the results section instead of the end. The background needed to understand the results is detailed in the last two paragraphs of the methods section. This is GIS data and it is explained in that section that we used a combination of ArcGIS software, the Network Analyst extension tool and routing algorithms to compare the driven routes to the actual routes. Our coauthor who is an expert in GIS, AA, indicates that unfortunately there are no p-values or confidence intervals to report with this type of data. Based on the GIS data captured from the COTS device, the 30 seconds was more accurate with fewer errors compared to the 60 and 120 seconds interval. The dataset is available for review.
